# Evaluation of the immunogenicity of a Crimean-Congo hemorrhagic fever virus vaccine candidate in mice developed based on a baculovirus Zera nanoparticle delivery system

**DOI:** 10.3389/fvets.2023.1126785

**Published:** 2023-06-01

**Authors:** Gang Zhang, Pu Wang, Lingling Jiang, Yunyi Kong, Sheng Wang, Yong Li, Sinong Zhang

**Affiliations:** ^1^School of Life Sciences, Ningxia University, Yinchuan, China; ^2^Key Laboratory of Ministry of Education for Conservation and Utilization of Special Biological Resources in Western China, Ningxia University, Yinchuan, China

**Keywords:** Crimean-Congo hemorrhagic fever, Zera nanoparticles, baculovirus expression system, vaccine, immunological evaluation

## Abstract

Crimean-Congo hemorrhagic fever (CCHF) is a zoonotic disease caused by Crimean-Congo hemorrhagic fever virus (CCHFV), which can cause severe clinical disease and even death in humans. In recent years, the disease has spread to a wider area, posing a major public health threat to China as well as the Middle East, Europe and Africa, and there is no safe and effective vaccine to prevent the disease. Recently, it has been shown that using the Zera fusion to target proteins can enhance immunogenicity and improve the potential for developing viral vaccines. Based on this finding, in this study, two vaccine candidates, Zera-Gn and Zera-Np, were prepared using an insect baculovirus system expressing CCHFV glycoprotein (Gn) and nucleocapsid protein (Np) fused with Zera tags, and evaluated for immunogenicity in BALB/c mice. The obtainedresults showed that both Zera-Gn and Zera-Np recombinant nanoparticles were successfully expressed, and Zera-Gn had good induction of humoral and cellular immunity in mice, and its immunogenicity was significantly higher than that of Zera-Np. The results indicated that Zera-Gn self-assembled nanoparticles prepared by fusing Zera tags with CCHFV spike-in protein Gn have the potential to be a candidate vaccine for CCHF, and this study provides a reference for the development of Zera self-assembled nanoparticle vaccine for CCHF.

## 1. Introduction

Crimean-Congo hemorrhagic fever (CCHF) is widely prevalent in several regions of Asia, Europe, and Africa and is transmitted to humans mainly by ticks, herbivorous livestock, and pets (1, 2). CCHF clinical symptoms are similar to those of typical influenza, manifesting as fever, diarrhea, fatigue, and drowsiness (3), and in severe cases, patients develop renal lesions, liver failure, and lung damage, with a mortality rate of approximately 40% in humans (4). There is no safe and effective vaccine to prevent this disease, and the virus poses a public health threat to many countries and regions as CCHFV is spreading over a wider geographic area (5).

Crimean-Congo hemorrhagic fever virus (CCHFV) is a member of the genus Nairovirus, family Bunyaviridae (6), which consists of three single-stranded negative-sense RNA fragments, L, M and S. The L fragment encodes an RNA-dependent RNA polymerase, the M fragment encodes a spike-in protein precursor (GPC), and the S protein encodes a nucleocapsid protein (Np) (7). The Gn and Gc proteins encoded by the M fragment are important antigens for the development of vaccine (8), and Gn and Gc neutralizing epitopes can bind target cells and affect viral infectivity (9). Gc induces the production of specific neutralizing antibodies in the host organism, but provides limited protection in mice, whereas some vaccines developed with Gn as antigen protect mice from CCHFV infection via passive immunization (10). In addition, the Np protein is an important antigen for vaccine development, and the S fragment is more conserved than the M fragment. This indicates that Np proteins produces a broader immune response when used as components of vaccine formulations (11), such as Np protein adenovirus vaccines (which protect mice from CCHFV challenge (12)) and Np protein mRNA vaccines (which protect some mice from CCHFV challenge (13)). Thus, both Gn and Np proteins of CCHFV can be used as protective antigens.

To date, the only vaccine developed is the inactivated vaccine developed in the former Soviet Union, which was administered in Bulgaria and subsequently discontinued due to its limited protective efficacy (14). In recent years, researchers have developed vaccines against various forms of CCHF, such as DNA vaccines (SAHİN et al. (15),), virus-like particle (VLP) vaccines (16), plant vector vaccines (17), subunit vaccines (18), modified vaccinia virus Ankara (MVA) vaccines (19), and mRNA vaccines (20). Some of these vaccines have also demonstrated their efficacy in experimental animals, but none have been evaluated in clinical trials. Among them, subunit vaccine research is relatively mature, with high preparation purity, few side effects, and high safety, and this approach has become one of the important strategies for developing CCHF vaccines (18).

In this study, we chose the baculovirus expression system, which is highly established in vaccine development; the application of this system has led to the approval of several clinical subunit vaccines. We added novel plant protein body (Zera) protein sequences to both ends of the target protein. Zera proteins can improve immunogenicity, which is a new strategy in current vaccine development; this strategy has been used for the bluetongue virus VP2 protein vaccine (21), the HPV E7 protein vaccine (22), and the influenza virus M2e protein vaccine (23). Zera^®^ (γ-Zein ER-accumulating domain) is a proline-rich structural domain consisting of 112 amino acids (24). The target protein can be induced to form dense spherical protein bodies of approximately 10–20 nm (25), which are retained in the endoplasmic reticulum, thus avoiding hydrolysis by proteases in the cytoplasm (22).

Therefore, in this study, we aim to express the CCHFV Np protein and Gn protein fused with Zera tags by baculovirus system, to evaluate the immunogenicity of the prepared recombinant nanoparticles as vaccine candidates in mice, and to provide a theoretical basis for the development of CCHF nanoparticle vaccines.

## 2. Materials and methods

### 2.1. Cell and viruses

Alfalfa silvery night moth (Sf9) cells (Invitrogen, United States) were cultured in serum-free Sf-900 II SFM medium (Gibco, Grand Island, NY, United States) at 27°C. The baculovirus expression vector pFastBac Dual (Invitrogen, United States) was provided by Prof. Yulong He from Zhejiang University of Technology. The wild-type baculovirus rvAc-dual was kept in our laboratory, and vector validation sequencing was performed by Jilin Kumi Biological Co.

### 2.2. Construction of recombinant baculoviruses

**Figure 1:**
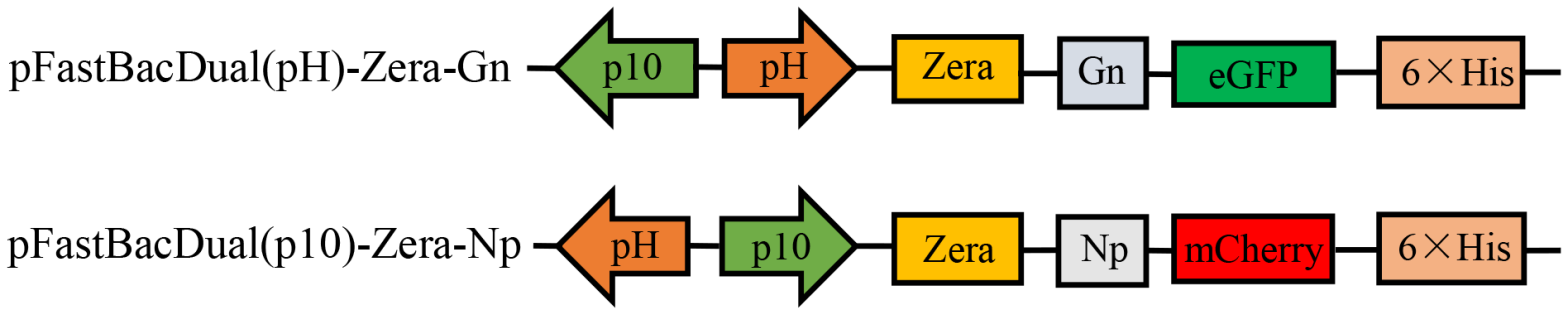
Recombinant plasmid mapping.

Based on previous studies and epidemiological surveys of ticks in Xinjiang and Inner Mongolia in our laboratory (26), we selected the glycoprotein Gn (284 aa) and nucleocapsid protein Np (483 aa) of the Chinese Xinjiang strain HANM-18 (MN832721) (27), and Zera (KU593570.1) sequences were added to the N-terminus of the Gn and Np sequences, with EGFP and mCherry used as fluorescent tags and His tags added at the C-terminus. The fusion construct Zera-Gn-mCherry-His was inserted into the pH end of pFast Bac Dual to obtain pFast Bac Dual (pH)-Zera-Gn-mCherry-His; Zera-Np-EGFP-His was inserted into pFast Bac Dual (p10)-Zera-Np-EGFP-His, which was obtained by inserting Zera-Np-eGFP-His into the p10 end of pFast Bac Dual (Figure 1).

### 2.3. Preparation and proliferation of recombinant baculoviruses

pFastBac Dual (pH)-Zera-Gn-mCherry, pFastBac Dual (p10)-Zera-Np-eGFP and pFastBac Dual, which were synthesized and validated by the sequencing company, were sequentially transformed into DH10 Bac receptor cells (Biomed, China), and positive colonies were obtained by blue–white spot screening. Positive colonies were inoculated into LB medium, and the bacmids were extracted and named Dual (pH)-Zera-Gn-mCherry, Dual (p10)-Zera-Np-eGFP and Dual. According to the user manual (TransIT-LT1 Transfection Reagent, Mirus), the bacmids were transfected into Sf9 cells, and the expression of fluorescent proteins at different time periods was observed by inverted fluorescence microscopy at 72 h, 96 h and 120 h after transfection.

### 2.4. Indirect immunofluorescence and electron microscopy observation

For observation of the localization of Zera-Gn and Zera-Np proteins in Sf9 cells, recombinant baculovirus was first added dropwise to Sf9 cells at an MOI = 0.1; indirect immunofluorescence experiments were conducted as previously reported (18), and the following antibodies were used in this experiment: mouse anti-His monoclonal antibody (1:500 dilution; Abcam) and the secondary antibodies CoraLite 488-labeled goat anti-mouse IgG (1:500 dilution; Proteintech) and CoraLite 594-labeled goat anti-mouse IgG (1:500 dilution; Proteintech). Laser confocal microscopy (Leica TCS SPE, Wetzlar, Germany) was used to observe the localization of fluorescence. To observe the microstructure of Zera nanoparticles in Sf9 cells, we sent infected Sf9 cells to Xavier for electron microscopy.

### 2.5. Western blotting assay for Zera nanoparticles

The larger molecular weight of the fluorescent tags may affect the spatial structure and function of proteins, which further affects the immune effect. Therefore, we modified the two vectors to produce proteins without fluorescent tags, named dual-Zera-Gn and dual-Zera-Np, and we prepared these nonfluorescent recombinant baculoviruses using the abovementioned method. Zera-Gn and Zera-Np nanoparticles were purified by sucrose density gradient centrifugation and identified by western blotting. The primary antibody was a mouse anti-His monoclonal antibody (1:5000 dilution; Abcam); the secondary antibody was a rabbit anti-mouse monoclonal antibody (1:5000 dilution; Proteintech).

### 2.6. Immunization experiments in mice

Thirty-five female BALB/c mice (7–8 weeks of age, purchased from Beijing Vitalihua Laboratory Animal Technology Co., Ltd., certificate of conformity No. SCXK JING 2021–0006) were randomly divided into five groups (*n* = 9). Sera were collected from the retro-orbital sinus at 0, 14, 28, 35 and 42 d after immunization (DAI) (Table 1); four mice from all immunized groups were randomly selected at 35 d and 42 d, their spines were dislocated, and splenocytes were isolated from their spleens for splenic lymphocyte proliferation experiments (Animal Ethics Committee, Experimental Animal Center, Ningxia Medical University).

**Table 1:** Immunization strategy in mice.

Group	Immunization time (DAI)	Immunization dose
1. Blank	–	–
2. PBS	0, 14, 28	100 μL
3. rvAc-dual	0, 14, 28	10^8^ PFU
4. Zera-Gn	0, 14, 28	10 μg
5. Zera-Np	0, 14, 28	10 μg

### 2.7. Analysis of total serum IgG and cytokines in mice

Antibody levels in serum were detected by enzyme-linked immunosorbent assays (ELISAs) as described previously (28), and the antigens used were the prokaryotic-expressed and purified antigens Gn and Np at a concentration of 0.1 mg/mL; primary antibodies were serum samples analyzed at 0, 14, 28, 35, and 42 d after the first immunization (diluted 1:100 with the blocking solution); the secondary antibodies were goat anti-mouse IgG labeled with horseradish peroxidase (HRP) (diluted 1:1000 with the blocking solution); and all ELISAs were performed in three replicates. The absorbance values at 450 nm were measured by enzyme standardization after termination of the reaction. TNF-α and IL-4 cytokines were detected in mouse sera by a commercial ELISA kit (Mouse IFN-γ/IL-4 ELISA BASIC kit Boster), and all sera to be tested were made in three replicates. The absorbance values were measured at OD450 nm using a spectrophotometer.

### 2.8. Mouse spleen lymphocyte experiment

Isolated splenocytes were plated in microtiter plates at 5 × 10^5^ cells/well, and 100 μL of stimulant (Gn and Np proteins) at a concentration of 2 μg/mL was added to each well; the negative control group was incubated at 37°C for 42 h. Then, 100 μL of RPMI 1640 (Gibco) medium was added to each well, and the positive control was incubated with 100 μL of knife bean protein A (5 mg/mL). Each well was incubated with 100 μL of DMSO, and the absorbance value was measured at 490 nm using an enzyme marker. The stimulation index (SI) was calculated for each group, i.e., negative control (RPMI-1640) mean OD_490_ nm/positive control (knife bean protein A, Solarbio) mean OD_490_ nm.

### 2.9. Statistical analysis

Statistical analysis was performed using GraphPad Prism® version 6 for Windows (GraphPad Software, San Diego, CA, United States). Data are expressed as the mean ± standard deviation. A Bonferroni *post hoc* test was used to compare the immune response among groups. Data were considered significantly different when *p* was <0.05.

## 3. Results

### 3.1. Construction and characterization of CCHFV Gn and np Zera nanoparticle vectors

**Figure 2:**
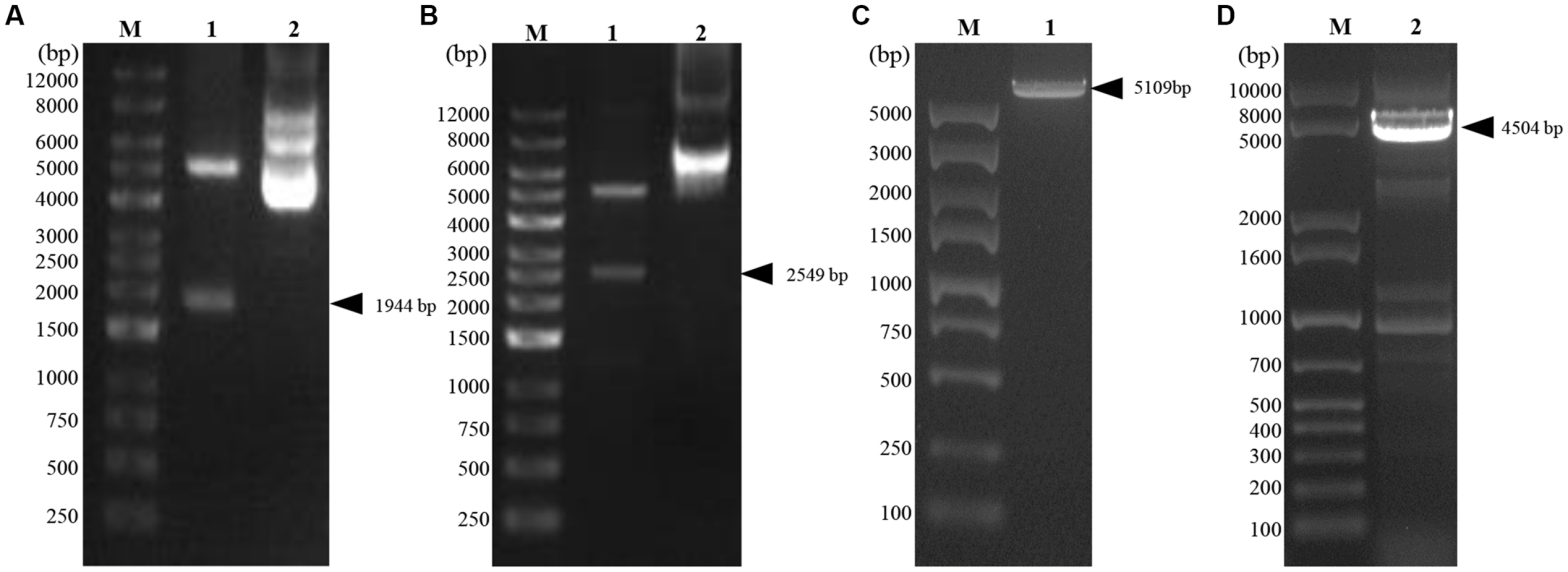
Identification of recombinant plasmids by double digestion and bacmid PCR. **(A)** Zera-Gn-mCherry double digestion identification map, Lane 1: Zera-Gn-pFastBac Dual/Xho I + Sph I (1940 bp), Lane 2: plasmid; **(B)** Zera-Np-eGFP double digestion identification map, Lane 1: Zera-Np-pFastBac Dual/BamH I + Xba I (2,549 bp), Lane 2: plasmid; **(C,D)** Recombinant bacmid PCR identification. Lane 1: Bacmid-Zera-Np-eGFP PCR; Lane 2: Bacmid-Zera-Gn-mCherry PCR.

Two recombinant vectors, pFast Bac Dual-Zera-Gn-mCherry and pFast Bac Dual-Zera-Np-EGFP, constructed in this experiment were identified by double digestion (Figures 2A,B). The vectors were transformed into DH10Bac cells after validation by sequencing, and amplification of the extracted recombinant bacmid was verified using M13 universal primers (Figures 2C,D). The recombinant baculoviruses prepared for transfection of Sf9 cells were named rvAc-Zera-Gn-mCherry and rvAc-Zera-Np-EGFP.

### 3.2. Expression and localization analysis of Zera-Gn and Zera-np nanoparticles

**Figure 3:**
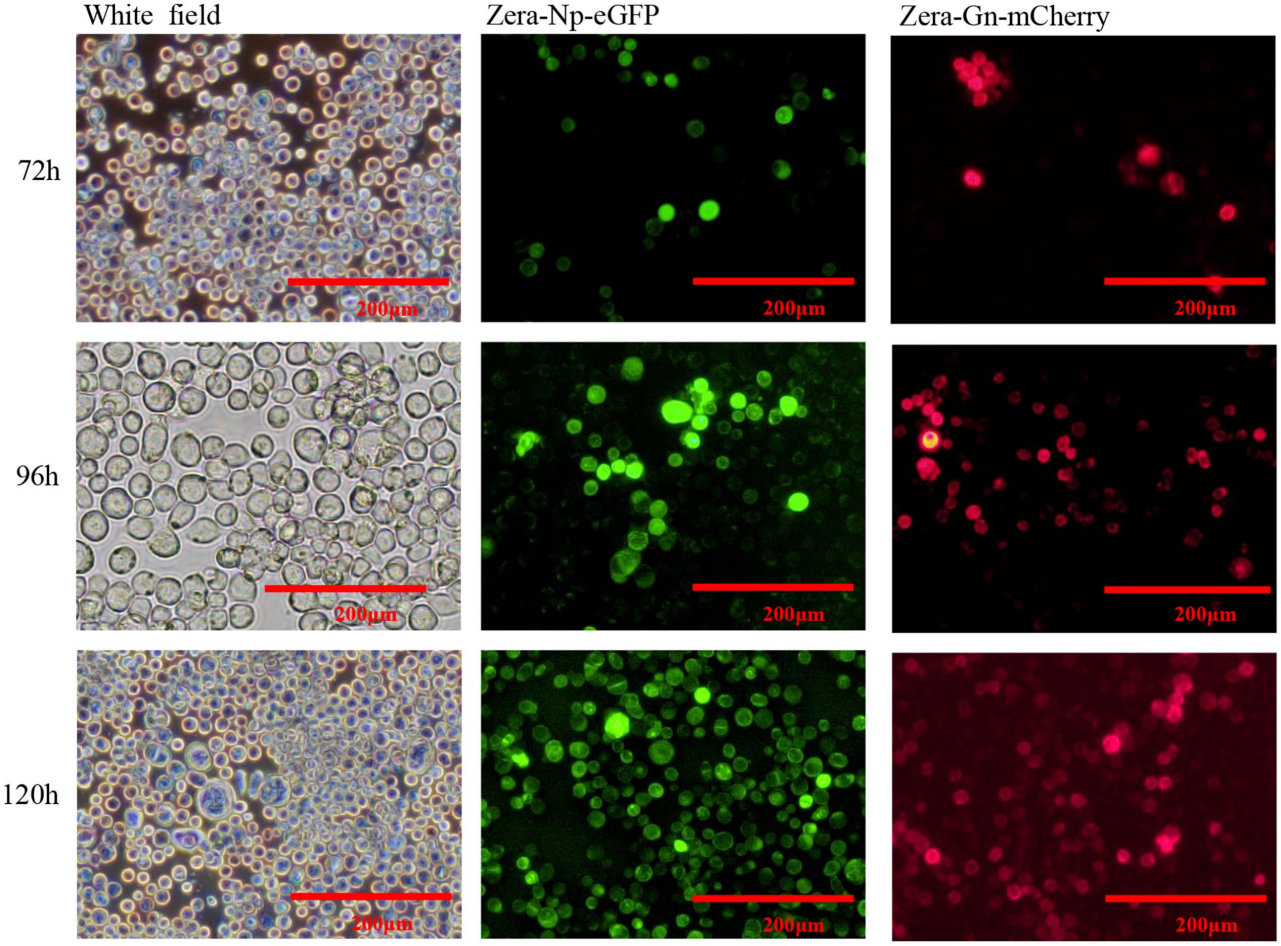
Direct fluorescence observation of Zera-Gn-mCherry and Zera-Np-eGFP recombinant bacmid transfected in Sf9 cells (72 h, 96 h and 120 h).

**Figure 4:**
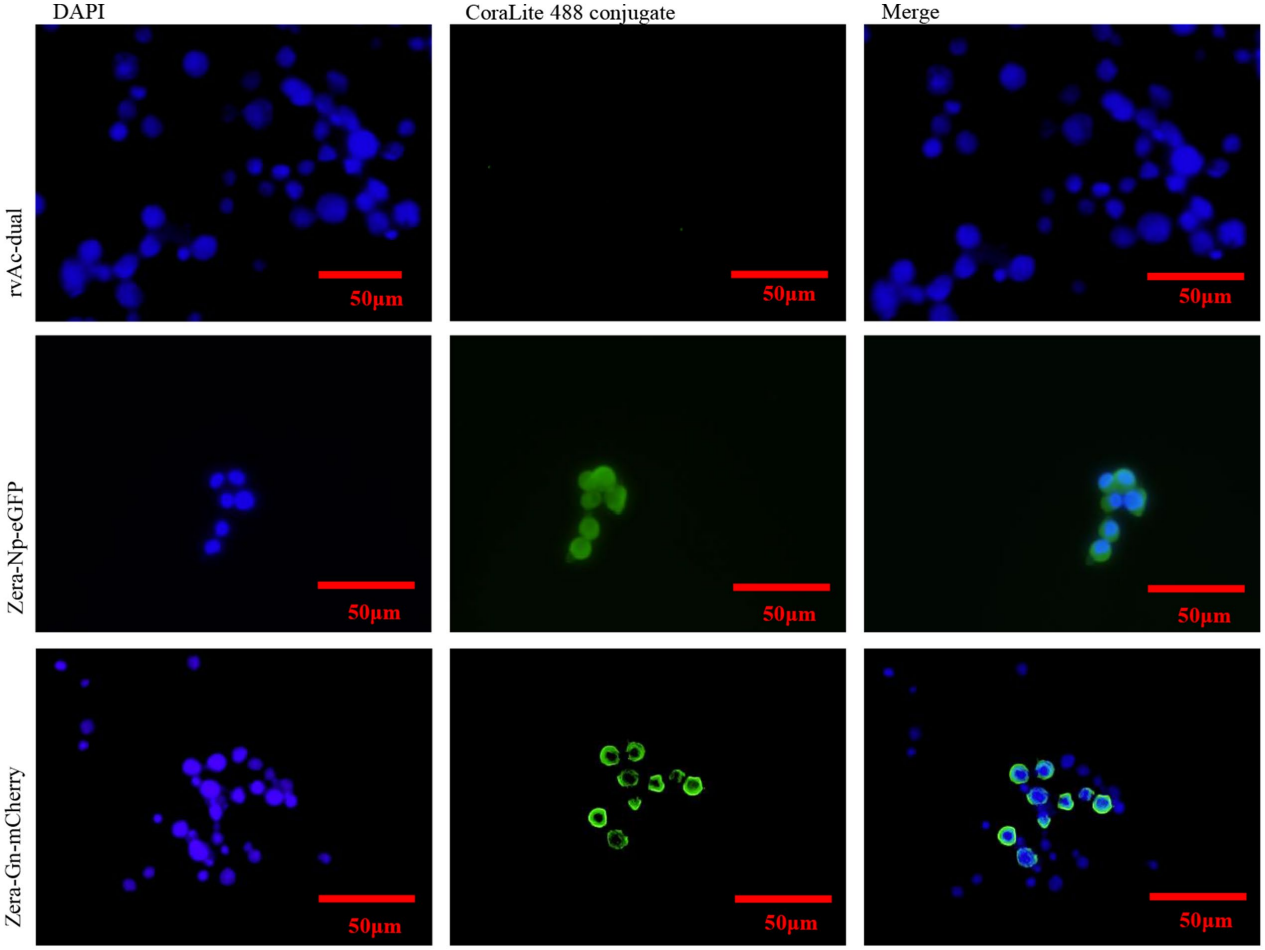
Indirect immunofluorescence identification of recombinant Zera-Gn and Zera-Np nanoparticles and wild-type viruses.

**Figure 5:**
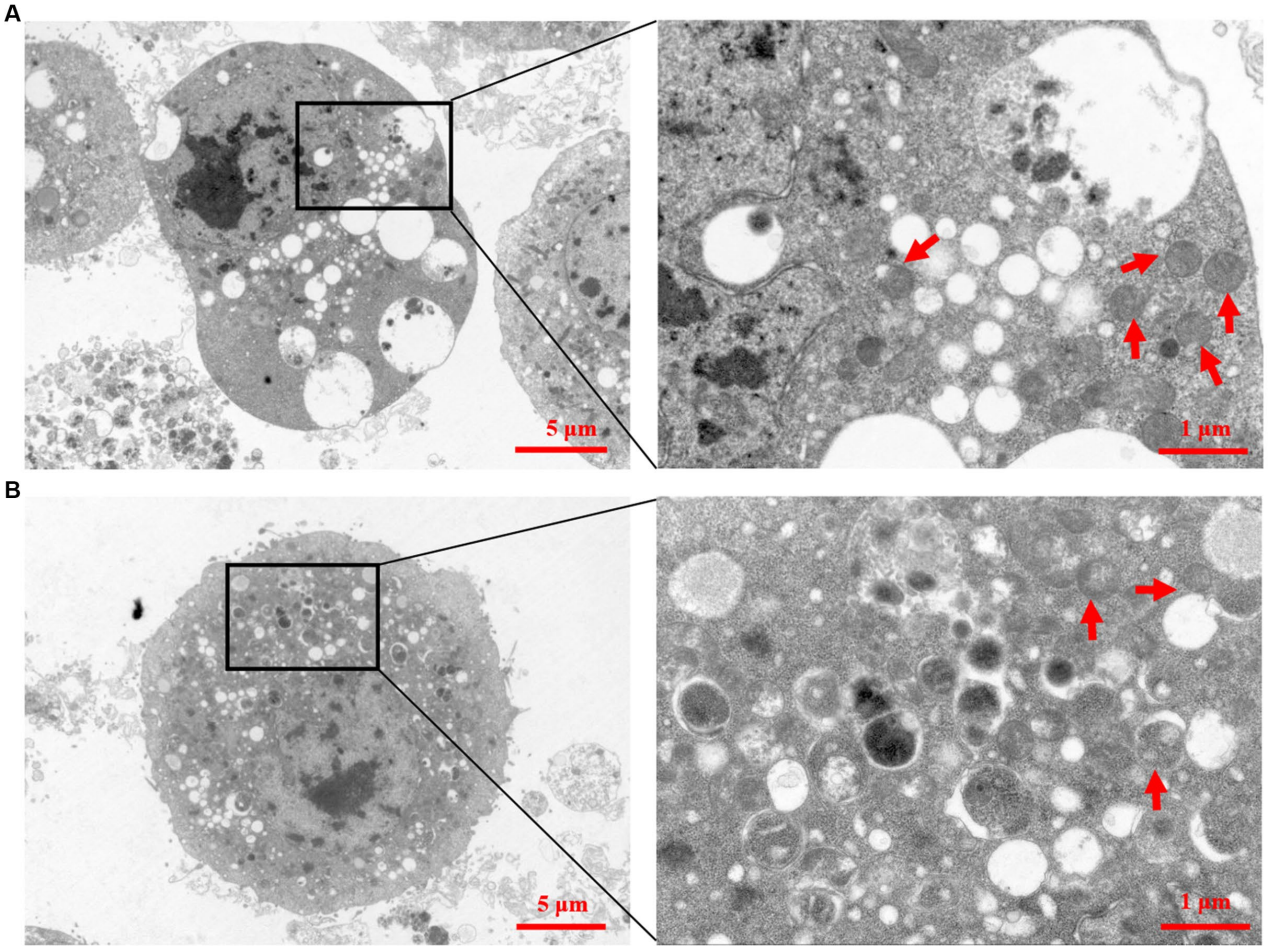
Transmission electron micrographs of recombinant Zera nanoparticles. **(A)** Transmission electron micrograph of recombinant Zera nanoparticles (Zera-Np). **(B)** Transmission electron micrograph of recombinant Zera nanoparticles (Zera-Gn).

Two recombinant baculoviruses, rvAc-Zera-Gn-mCherry and rvAc-Zera-Np-eGFP, were transfected into Sf9 cells, and the expression of fluorescent proteins in Sf9 cells at 72, 96 and 120 h after transfection was observed by inverted microscopy at different excitation wavelengths (white, green, blue and green light; Figure 3). To determine the localization of the Gn and Np proteins in the cells, we performed indirect immunofluorescence and observed by laser confocal microscopy that both Zera-Gn-mCherry and Zera-Np-eGFP were localized near the cytoplasmic membrane (Figure 4). Furthermore, we observed the localization of Zera-Gn and Zera-Np nanoparticles in Sf9 cells by transmission electron microscopy and confirmed the formation of 0.4–1.1 μm protein body organelles in the cytoplasm (Figure 5).

### 3.3. Recombinant Zera nanoparticles induce immune responses in mice

**Figure 6:**
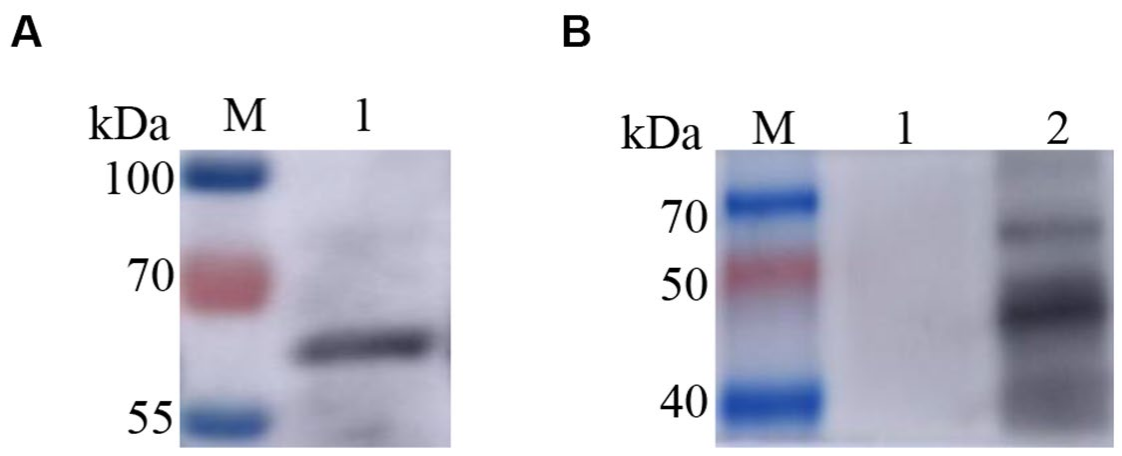
Western blotting identification of recombinant Zera nanoparticles. **(A)** western blotting identification of Zera-Np nanoparticles (67.2 kDa); **(B)** Lane 1: negative control rvAc-dual, Lane 2: western blot identification of Zera-Np nanoparticles (45.2 kDa).

**Figure 7:**
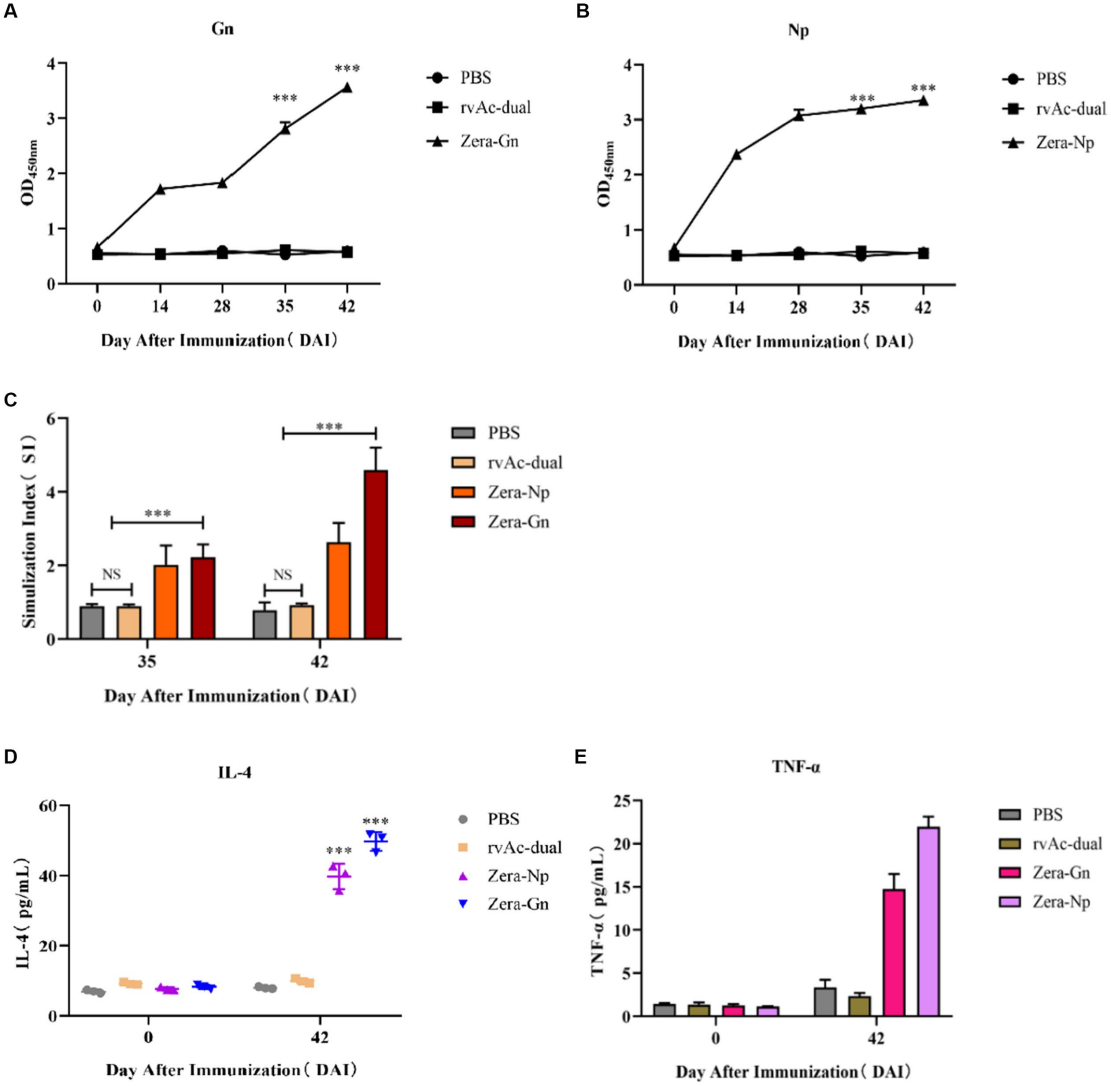
Analysis of immunogenicity in mice. Analysis of induced IgG responses in mice by indirect ELISA for the Gn recombinant protein **(A)** and the Np recombinant protein **(B)**. The y-axis represents the OD450 nm of serum samples collected at 0, 14, 28, 35 and 42 d after immunization (DAI) for each group, ^***^p < 0.001, with significant differences from PBS and rvAc-dual (Bonferroni test). **(C)** Results of lymphocyte proliferation experiments. The y-axis represents the stimulation index of splenic lymphocyte samples collected at 35 and 42 DAI. ns: no significant difference; ^***^p < 0.001, significant difference (Bonferroni test). Quantitative analysis of IL-4 **(D)** and TNF-α **(E)** levels in the serum of immunized mice. All analyses were performed in triplicate, and error bars show the standard deviation (SD).

Two recombinant vectors, pFast Bac Dual-Zera-Gn-mCherry and pFast Bac Dual-Zera-Np-EGFP, were modified by removing the fluorescent label to prepare nonfluorescent Zera-Gn and Zera-Np nanoparticles, and western blotting was used to detect the expression of the resultant Zera nanoparticles (Figure 6). To investigate the humoral immune response to Zera-Gn and Zera-Np nanoparticles, total IgG levels in the sera of immunized mice at 0 d, 14 d, 28 d, 35 d and 42 d were measured using an indirect ELISA method. The results showed that the total IgG levels of Zera-Np and Zera-Gn nanoparticles were significantly higher than those in the negative control group at 14 d, 35 d and 42 d (Figures 7A,B), and the total IgG content of the Zera-Gn group was higher than that in the Zera-Np group after three immunization doses. To investigate the cellular immune response to the recombinant baculoviruses in mice, we examined the SI of spleen lymphocytes of immunized mice at 35 and 42 d with different stimulating agents (Figure 7C). The SI values of the Zera-Gn and Zera-Np groups were significantly higher than those of the PBS and wild-type virus groups at 14 d. The SI value of Zera-Gn was significantly higher than that of Zera-Np at 42 d (*p* < 0.001).

To further assess cellular immunity levels, we analyzed the changes in serum levels of IL-4 and TNF-α in mice (Figures 7D,E), and the obtained results showed that the serum levels of IL-4 and TNF-α in both Zera-Gn-and Zera-Np-treated mice were significantly higher than those in the immune control mice (*p* < 0.001). The serum levels of IL-4 and TNF-α were 49.697 ± 2.732 pg./mL and 21.974 ± 1.159 pg./mL in mice in the Zera-Gn group, respectively, and the serum levels of IL-4 and TNF-α in mice in the Zera-Np group at 42 d were 39.712 ± 3.599 pg./mL and 14.732 ± 1.750 pg./mL, respectively.

## 4. Discussion

In recent years, with the rapid development of molecular biology as well as bioinformatics, researchers have developed various CCHF vaccine candidates (29), but there is no internationally recognized, safe and effective CCHF vaccine due to the lack of suitable animal models and secure biological laboratories needed to confirm their protective properties (30). Therefore, the development of a CCHF vaccine is an important topic.

In this study, we fused the plant protein (Zera) to the N-termini of Gn and Np of CCHFV for the first time, expressed recombinant nanoparticles using the Bac-to-Bac system, and evaluated the immunogenicity of these constructs in BALB/c mice. The Bac-to-Bac expression system allows the target protein to have multiple eukaryotic modifications (13); the fusion of the Zera tag with the target protein can enhance the expression of the target protein in insect cells, and the Zera tag can act as a molecular adjuvant to enhance the immunogenicity of the antigen in animals (31). In this study, both the Zera-Gn and Zera-Np proteins were successfully expressed in the Bac-to-Bac system, and indirect immunofluorescence experiments showed that the Gn and Np proteins were retained in the cytoplasm of Sf9 cells after expression as a fusion protein with Zera; this behavior was similar to that of Zera in other cells, which is nontoxic, has good stability and can increase protein accumulation and provide protection against degradation by host cell enzymes (32), thus providing high-quality material for later animal immunization experiments. Transmission electron microscopy observations further demonstrated that both Zera-Gn and Zera-Np could form nanoparticles (0.4–1.1 μm in diameter) in Sf9 cells.

After immunization of mice, IgG antibody levels were significantly higher in the Zera-Gn and Zera-Np groups than in the control group, indicating that both nanoparticles could induce a significant humoral immune response in mice (*p* < 0.001). Splenic lymphocyte proliferation experiments showed that the SI was significantly higher in the Zera-Gn and Zera-Np groups (*p* < 0.001) and significantly higher in the Zera-Gn group than in the Zera-Np group (*p* < 0.001), indicating that the Gn protein induced production of significantly higher levels of cellular immunity than the Np protein (Figures 7D,E). This observation is in line with other studies that reported similar results (33), which may be because Gn is derived from the CCHFV stinger protein, whereas the Np protein is an intracellular protein; the stinger protein first interacts with the host cell receptor during viral invasion, mediating the entry of the virus into the cell and acting as a target for neutralizing antibodies, which elicits a stronger cellular immune response in the organism.

T-cell immunity is associated with the clearance of pathogens (34), and in CCHF survivors, CD4+ effector cells are mainly of the Th1 type (34, 35). These cells secrete TNF-α, which activates neutrophils to phagocytose and digest pathogens, promoting cellular immune function (36); the secretion of IL-4 by Th2-type cells induces specific antibody production. The IL-4 and TNF-α in the sera of Zera-Gn-and Zera-Np-treated mice after three immunization doses were significantly higher than those in the control group (*p* < 0.001), with up to 49.69 pg./mL IL-4 and 21.974 pg./mL TNF-α in the serum of the Zera-Gn group mice; these results indicated that significant Th1-and Th2-type cellular immune responses were induced (*p* < 0.001), with a dynamic balance between the Th1 type involved in protection against the organism and the Th2-type cellular immune response that contributes to the clearance of the infection (37). These findings indicated that recombinant nanoparticles fused with Zera tags induce significant humoral and cellular immunity in mice and that the immunogenicity of Zera-Gn is much higher than that of Zera-Np. Due to the lack of a suitable commercial vaccine, the immunogenicity of the Zera-Gn construct cannot be compared with that of commercial vaccines, but future challenge experiments in BL4 laboratories with higher biological safety grade are needed to assess its value as a vaccine candidate.

## 5. Conclusion

In conclusion, we prepared two types of nanoparticles, CCHFV Zera-Gn and Zera-Np, and evaluated their immunogenicity in mice. The obtained results showed that Zera-Gn was significantly more immunogenic than Zera-Np and induced stronger humoral and cellular immunity in mice, indicating that Zera-Gn is a potential vaccine candidate for CCHF; further challenge experiments are needed to determine its protective properties.

## Data availability statement

The raw data supporting the conclusions of this article will be made available by the authors, without undue reservation.

## Ethics statement

The animal study was reviewed and approved by Ningxia Medical University.

## Author contributions

GZ carried out experimental work and wrote the manuscript. PW crunched the numbers. LJ conducted gene design and culture of Sf9 cells. YK cultured Sf9 cells. SW and SZ provided technical guidance. YL provided the initial idea, designed the study, and revised the manuscript. All authors contributed to the article and approved the submitted version.

## Funding

This work was supported by the National Natural Science Foundation of China (grant number: 32130104), the Key Research, Development Program of Ningxia Hui Autonomous Region (grant number 2021BEF02028), and the introduced talents special project for key research development program of NingXia (2022BSB03093).

## Conflict of interest

The authors declare that the research was conducted in the absence of any commercial or financial relationships that could be construed as a potential conflict of interest.

## Publisher’s note

All claims expressed in this article are solely those of the authors and do not necessarily represent those of their affiliated organizations, or those of the publisher, the editors and the reviewers. Any product that may be evaluated in this article, or claim that may be made by its manufacturer, is not guaranteed or endorsed by the publisher.
